# The neurocognitive functioning in bipolar disorder: a systematic review of data

**DOI:** 10.1186/s12991-015-0081-z

**Published:** 2015-12-01

**Authors:** Eirini Tsitsipa, Konstantinos N. Fountoulakis

**Affiliations:** Aristotle University of Thessaloniki, Thessaloniki, Greece; Division of Neurosciences, 3rd Department of Psychiatry, School of Medicine, Aristotle University of Thessaloniki, 6, Odysseos street (1st Parodos, Ampelonon str.) 55536 Pournari Pylaia, Thessaloniki, Greece

## Abstract

**Background:**

During the last decades, there have been many different opinions concerning the neurocognitive function in Bipolar disorder (BD). The aim of the current study was to perform a systematic review of the literature and to synthesize the data in a comprehensive picture of the neurocognitive dysfunction in BD.

**Methods:**

Papers were located with searches in PubMed/MEDLINE, through June 1st 2015. The review followed a modified version of the recommendations of the Preferred Items for Reporting of Systematic Reviews and Meta-Analyses statement.

**Results:**

The initial search returned 110,403 papers. After the deletion of duplicates, 11,771 papers remained for further evaluation. Eventually, 250 were included in the analysis.

**Conclusion:**

The current review supports the presence of a neurocognitive deficit in BD, in almost all neurocognitive domains. This deficit is qualitative similar to that observed in schizophrenia but it is less severe. There are no differences between BD subtypes. Its origin is unclear. It seems it is an enduring component and represents a core primary characteristic of the illness, rather than being secondary to the mood state or medication. This core deficit is confounded (either increased or attenuated) by the disease phase, specific personal characteristics of the patients (age, gender, education, etc.), current symptomatology and its treatment (especially psychotic features) and long-term course and long-term exposure to medication, psychiatric and somatic comorbidity and alcohol and/or substance abuse.

## Background

The neurocognitive dysfunction in BD has been the focus of debate for many years. It was not clear whether the observed neurocognitive deficit could be explained by iatrogenic or alcohol and/or drug abuse effects or by the temporary functional changes which constitute the result of mood changes. Also, it was unclear whether the impairment is the product of degenerative structural brain changes or of some kind of structural changes of a neurodevelopmental origin (trait), or it is secondary to mood dysregulation (state). Recent data suggested that the neurocognitive deficit is not only an enduring component of the illness, but also represents a core primary characteristic of the illness, rather than being secondary to the mood state or medication [1]. It has been suggested by recent data that 84 % of patients suffering from schizophrenia, 58.3 % of psychotic major depressive patients, and 57.7 % of psychotic BD patients are neurocognitively impaired (at least one SD below healthy controls in at least two domains) [2].

It has also been suggested that patients with BD are more creative (e.g., artists, scientists, etc.) and have higher IQ in comparison to the general population [3–7]. However, more recent data reported a significant and broad neurocognitive deficit, which seems to be present even before the first manifestation of mood symptoms, and it persists across the different phases and even worsens during the course of the illness [8–13]. Several studies suggest that 40 % of BD patients are impaired in one neurocognitive domain, one-third or more are impaired in at least two neurocognitive domains and 22 % in three or more domains [14, 15]. This deficit is rather stable and relatively independent from mood changes, probably reflecting trait features of BD [16–19] Even after controlling for confounding variables, such as education and social class and clinical symptoms, it has been indicated that the neurocognitive impairment in BD is less pronounced in comparison to that in schizophrenia [20, 21].

Gender, age, education, phase of the illness and medication status constitute some of the identified confounding factors. Additionally, patients in a severe depression or mania cannot be tested.

A significant limitation in this kind of research is that the performance in most tests is influenced by more than one neurocognitive process. It is a fact that the boundaries between neurocognitive processes are unclear and no process is completely independent from the others. Different approaches in their classification and nomenclature have been proposed, adding to the confusion. The domain of executive functions, particularly, is open to several different approaches and conceptualizations.

The aim of the current study was to perform a systematic review of the literature to identify all studies pertaining to the topic of neurocognitive dysfunction in BD and afterwards to synthesize the findings in a comprehensive picture.

## Review

### Materials and methods

Reports investigating the neurocognitive dysfunction in BD patient samples were located with searches in Pubmed/MEDLINE, through June 1st 2015. Only reports in English language were included.

The Pubmed database was searched using the search terms ‘Bipolar’ OR ‘Mania’ OR ‘manic’ OR ‘Manic-depression’ OR Manic-depressive AND ‘Neurocognition’ OR ‘Neurocognitive’ OR ‘Neuropsychology’ OR ‘Neuropsychological’ OR ‘Cognitive’ OR ‘Cognition’ OR ‘Intelligence quotient’ OR ‘IQ’ OR ‘VIQ’ OR ‘PIQ’ OR ‘North American Adult Reading Test’ OR ‘NAART’ OR ‘wide Range Achievement Test’ OR ‘WRAT’ OR ‘Wechsler Adult Intelligence Scale’ OR ‘WAIS’ OR ‘Mental speed’ OR ‘Digit Symbol Substitution Test’ OR ‘DSST’ OR ‘Trail Making Test’ OR ‘TMT’ OR ‘Reaction time’ OR ‘Attention’ OR ‘Attentional’ OR ‘Vigilance’ OR ‘Concentration’ OR ‘Continuous Performance Test’ OR ‘CPT’ OR ‘Digits Forward’ OR ‘Learning’ OR ‘Memory’ OR ‘Working memory’ OR ‘Declarative memory’ OR ‘Verbal memory’ OR ‘Non-verbal memory’ OR ‘Visual memory’ OR ‘Logical memory’ OR ‘Autobiographical memory’ OR ‘Prospective memory’ OR ‘Immediate memory’ OR ‘Delayed memory’ OR ‘Verbal learning’ OR ‘Digits Backward’ OR ‘California Verbal Learning Test’ OR ‘CVLT’ OR ‘Rey Auditory Verbal Learning Test’ OR ‘RAVLT’ OR ‘Wechsler Memory Scale’ OR ‘WMS’ OR ‘Free recall’ OR ‘Rey Complex Figure Test’ OR ‘RCFT’ OR ‘Verbal skills’ OR ‘Verbal fluency’ OR ‘Category fluency’ OR ‘Letter fluency’ OR ‘Controlled Oral Word Association Test’ OR ‘COWA-FAS’ OR ‘Animal Naming’ OR ‘Visuospatial’ OR ‘Constructional’ OR ‘Block design’ OR ‘Rey Complex Figure Test’ OR ‘RCFT’ OR ‘Clock test’ OR ‘Executive function’ OR ‘Reasoning’ OR ‘Inhibitory control’ OR ‘Executive control’ OR ‘Concept formation’ OR ‘Wisconsin Card Sorting Test’ OR ‘WCST’ OR ‘Stroop Color Word Test’ OR ‘Stroop’ OR ‘SCWT’ OR ‘Theory of Mind’ OR ‘ToM’ OR ‘Emotion processing’ OR ‘Emotional decision-making’ OR ‘Benton Facial Recognition Test’ OR ‘BFRT’ OR ‘Faces Test’ OR ‘Eyes Test’ OR ‘Hinting Task’ OR ‘False belief and deception’ OR ‘Picture sequencing’ OR ‘Character intention’ OR ‘Faux Pas’.

Only papers in English language were included.

This review followed a modified version of the recommendations of the Preferred Items for Reporting of Systematic Reviews and Meta-Analyses (PRISMA) statement [22].

### Results

**Fig. 1:**
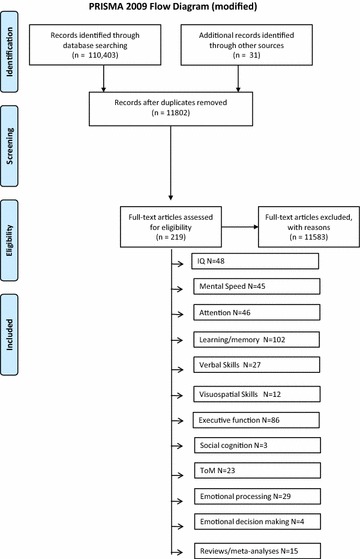
The PRISMA flowchart

The initial search returned 110,403 papers. Αfter the deletion of duplicates 11,771 remained. The reference lists of review papers and books were scanned and eventually and after assessing these papers on the basis of title and abstract 250 papers remained for further study (Fig. 1).

A list of neurocognitive domains and the neuropsychological tools usually used for their assessment are shown in Table 1.

**Table 1:** Neurocognitive domains assessed in the literature and neuropsychological tools used

Domain	Tool
Premorbid IQ	Single-word reading score from the North American Adult Reading Test (NAART)
	Wide Range Achievement Test (WRAT)
	Vocabulary subtest score from the Wechsler Adult Intelligence Scale (WAIS)
Current IQ	Wechsler Adult Intelligence Scale (WAIS)
Psychomotor and mental speed	Digit Symbol Substitution Test (DSST)
	Trail Making Test-A (TMT-A)
	Reaction time tests
Attention	Continuous Performance Test (CPT)
	Digits forward
Working memory	Digits backward
Verbal memory	
Learning	California Verbal Learning Test (CVLT)
Short delayed recall	Rey Auditory Verbal Learning Test (RAVLT)
Long delayed recall	Wechsler Memory Scale-Logical Memory (WMS-LM)
Recognition	Free recall
Non-verbal Memory	Rey Complex Figure Test (RCFT)—Immediate and delayed recall
	Wechsler Memory Scale-Visual Reproduction (WMS-VR)
Visuospatial function	Block design
	Rey Complex Figure Test (RCFT)-copy
Language/verbal fluency	Controlled Oral Word Association Test (COWA-FAS)
	Animal naming (AN)
Executive function	Wisconsin Card Sorting Test (WCST)—categories achieved and perseverative errors
	Stroop Color Word Test (SCWT)
	Trail Making Test-B (TMT-B)

#### General neurocognitive functioning and Intelligence quotient (IQ)

It has been reported that both patients with BD and their families have above average IQ and general intellectual functioning [23–29], or at least they have intelligence similar to healthy controls [30–34]. On the other hand, higher functioning and preserved neurocognitive performance during the premorbid phase, as well as higher social class could push towards a mood disorders diagnosis rather than schizophrenia [25, 30, 34–45].

Overall, the literature reports that patients with BD manifest moderate global reduction in their neurocognitive functioning as reflected in their IQ scores and their performance in neuropsychological batteries, irrespective of illness phase [43, 44, 46–48]. It has also been shown that these deficits are milder but may be qualitatively similar to those seen in patients with schizophrenia [21, 49]. The impairment is more severe in the presence of psychotic features [50], and, in line with this, it has been suggested that the deficit is comparable to that seen in schizophrenia, especially during the acute psychotic manic phase [51–54].

One study suggested the presence of a more complex relationship between BD and IQ since it has been reported that although BD was related to higher premorbid IQ, further analysis revealed that men with the lowest and the highest IQ (especially verbal or practical ability) were at the greatest risk for pure BD (without comorbidity) [55]. There are inconclusive data concerning whether psychotic BD is related to impaired premorbid IQ [56, 57].

It is well known that the full IQ is composed of two composite scores; the Verbal IQ (VIQ) and the non-verbal or Performance IQ (PIQ). The effect size of the deficit during the manic phase is 0.06 and 0.28 for PIQ and VIQ, respectively [53]. It seems that during acute mania the effect size is equal to 0.47 for the full IQ [54]. There are no studies concerning the IQ in patients during the acute depressive phase. Since it is possible, the premorbid IQ of BD patients is higher in comparison to the normal population, the true magnitude of decline cannot be evaluated accurately, and all studies reporting this decline in comparison to population norms underestimate it.

According to a meta-analysis, patients with BD show higher VIQ in comparison to PIQ scores [58, 59]. This is due to the uniform reduction in all PIQ subtest scores with an accompanying preservation of VIQ scores. This impairment is not because of the slowing in mental speed which is observed in BD patients (since four PIQ subtests in comparison to one VIQ subtest are timed) and this is obvious from the quality of the deficit which persists during periods of euthymia, and it is confirmed from targeted research [60]. The deficit might be already present during the early stages of BD [61] although more recent data argue against this [62]. One study has reported that the severity of depression seems to reduce VIQ scores and thus it might diminish the VIQ-PIQ discrepancy [63]. It is important to notice that this discrepancy does not seem to be present in patients with unipolar depression [64].

It has been reported that the VIQ-PIQ discrepancy might reflect a specific effect of BD on ‘fluid intelligence’ (the capacity to think logically and solve problems in novel situations, independently of acquired knowledge) with a simultaneous respect of the ‘crystallized intelligence’ (the ability to use skills, knowledge, and experience; it does rely on long-term memory). However, any attempt for a deep understanding of this deficit is risky and premature [63].

When all phases of the illness are taken into consideration, the effect sizes concerning current IQ reduction range from 0.36 to 070 [63, 65]. For patients in remission, the results of meta-analyses are inconclusive. The effect sizes reported for PIQ range from 0.40 to 0.50 [63, 66] to lower and within the normal range, that is 0.11–0.16 [8, 12]. A problem is that most meta-analyses report an effect size for the full IQ and not separately for VIQ and PIQ. The effect size of premorbid IQ change in euthymic BD patients is reported to be low and not significant (0.04–0.20) [66, 67].

#### Psychomotor and mental speed

Although mental speed and psychomotor activation are two concepts which overlap and include reaction time, cognitive and motor speed and, manual dexterity, they are clearly not identical. Additionally, most of the neuropsychological tools which are used for the evaluation of psychomotor and mental speed also assess other neurocognitive functions, and this is at least partially a consequence of a methodology effect, because to measure ‘speed’, you need to initiate a ‘procedure’ whose ‘speed’ is going to be measured.

It has been reported that the reaction time in bipolar depressive patients is prolonged [68]. Also, euthymic patients had not only prolonged reaction times but also more error rates in a visual backward masking test. Overall, reaction time prolongation has been found to be associated with burden of illness and especially past history of depressions but not with current medication [69].

Irrespective of illness phase and symptom severity, the speed of mental processing in BD patients seems to be slower [34, 43–45, 59, 70–87]. Moreover, it has been reported that the speed of mental processing is less affected in comparison to schizophrenia [38, 40, 61, 88], although there are some data suggesting the presence of a similar degree of impairment between patients with BD and patients with schizophrenia [89]. On the other hand, it has been suggested that there were no significant differences between depressed BD patients, euthymic BD patients and healthy controls in psychomotor speed [90]. Another study suggested that patients with BD showed a linear improvement in processing speed in the first year after resolution of their initial manic episode [91]. It has been reported that bipolar depressives manifest slower mental speed in comparison to manic and unipolar depressives, even after corrected for motor speed. The interesting feature in that particular study was that distraction improved the mental speed of BD depressed patients while it adversely influenced the speed of the other two groups [92].

The deficit could be present already during the early stages of BD [93]. Individual studies suggested that the magnitude of mental speed impairment in patients with BD is reported to correspond to an effect size of 0.82–1.08 [48, 77, 94]. When all phases of the illness are taken into consideration, the effect size 0.50–0.55 (which is similar to that observed concerning the IQ) [63, 65]. In euthymic BD patients, one meta-analysis reported that the effect size with the use of the TMT-A was 0.52 and with the DSST was 0.59 [13]. A second study reported effect sizes of 0.60 and 0.79, respectively [67], a third one reported effect sizes equal to 0.64 and 0.76 [12], a fourth 0.71 and 0.84 [8] and a fifth 0.7 and 07–0.8, respectively [66].

It is important to note that the deficit in the processing speed might have a significant confounding effect on the performance in almost all neurocognitive testing and controlling for it might make any difference between groups concerning other neurocognitive domains disappear [95]. Also, it is suggested that global functional impairment is strongly associated with poor performance on a cognitive measure of processing speed (e.g., WAIS Digit Symbol or the TMT) [75, 96].

#### Attention

Attention is a concept that includes a number of processes which work together, influence one another or prerequisite one another. These processes are: working memory (which refers to the ability to keep a limited number of mental objects in awareness for a limited duration of time), vigilance (which is the capacity to identify a specific target among many other stimuli), freedom from distraction or interference and the ability to split or to rapidly shift attention. Concentration is a term which refers to the ability to sustain attention over prolonged periods of time. There are many tests, with each of them assessing one of the previously mentioned processes. For example, the Continuous Performance Test assesses vigilance while ‘span tasks’ assess working memory. However, all these tests except from the specific aspect of attention they assess are also influenced from the other processes which are related to attention as well. Working memory is often classified as belonging to the executive functions and it is often considered in relation to them.

The magnitude of the attentional impairment is independent of current symptomatology and of the phase of the illness; however, significant variability is present in the literature [34, 44, 48, 71, 85, 87, 91, 97–113]. It has been also reported that the impairment is present already during the early stages of the disorder [93] and it is less pronounced in comparison to the deficit seen in patients with schizophrenia [38, 114, 115]. On the other hand, however, some studies reported that there is a similar magnitude of impairment between patient with BD and patients with schizophrenia [89, 116, 117]. It has been suggested that the performance in divided attention (DA) varied considerable over time within patients. It was also found a significant quadratic relationship between manic symptoms and DA performance, even after corrected for the effect of psychotropic medication. It has been suggested that mild hypomanic symptoms have a positive influence on divided attention scores and moderate to severe manic symptoms have a negative influence. No association between depressive symptoms and DA performance was found [118]. The magnitude of effect sizes ranges from 0.36 to 0.82 [48, 94, 119] depending on the domain assessed and the composition of the study sample.

The overall effect size calculated after a meta-analysis is 0.64 which is similar to that reported concerning the rest of neurocognitive functions [63]. Another meta-analysis reported that attention is impaired during the acute manic/mixed state, with effect sizes ranging from 0.79 to 0.90, during the acute depressed state with an effect size up to 0.80, but also during euthymia with effect size from 0.41 to 0.65 [11]. Another meta-analysis concerning BD patients during euthymia has reported medium effect sizes (0.48–0.60 depending on the testing condition) [13], while a third one found effect sizes of similar magnitude (0.62 and 0.74 for CPT hits and reaction time, respectively) [67]. A fourth meta-analysis gave effect size 0.58 for CPT and 0.37 for digits forward [8] and a fifth reported an effect size equal to 0.8 for CPT [66].

There are also a limited number of studies which indicate no impairment in attention [79, 120–123], and this concerns especially euthymic patients [124]. One study has found that depressed BD patients, euthymic BD patients and healthy controls had no significant differences in attention [90].

#### Learning and memory

Learning refers to the ability to acquire and store new information. Memory is the mental process that allows individuals to retrieve the new information at a later time. Learning and memory involve a number of processes including attention and concentration, encoding and allocation of effort. These processes are distinct from one another but interrelated and interdependent. Moreover, there are different strategies and processes involved, depending on whether a short- or a long-term effect is desirable and also depending on the quality and nature of the information and the frame it is presented in.

A typical classification of learning and memory structure is shown in Table 2 [125]. Due to the fact that much of research on memory is focused on ‘depression’ and does not distinguish between unipolar and bipolar depression, the results and the conclusions from these studies should be received with reservation because it is uncertain whether they apply specifically to BD, and to which extent.

**Table 2:** Effect sizes concerning the various neurocognitive domains during different phases of BD as well as in high-risk relatives (endophenotypes)

Domain	All phases	Acute mania	Acute bipolar depression	Euthymia	Endophenotype
Intelligence Quotient (IQ)					
Premorbid IQ	Normal				Normal
Current IQ	0.36–0.70	028–0.47		0.11–0.50	0.20
Psychomotor and mental speed	0.50–0.55			0.52–0.80	0.17–022
Attention	0.64	0.79–0.90	0.80	0.41–0.80	0.18–0.36
Memory					
Working memory	0.60			0.54–1.02	
Verbal memory					
Immediate	0.43			0.73–0.82	0.33–0.42
Delayed	0.34	1.05	1.20	0.71–0.85	0.27–0.33
Verbal learning	0.91	1.43		0.66–0.90	0.28
Non-Verbal memory					
Immediate	0.26			0.73	
Delayed	0.51			0.62–0.80	0.13
Episodic memory				0.62	
Visuospatial function	0.65			022–0.57	
Language/verbal fluency	0.63	0.51–0.59	0.93	0.34–0.90	0.27
Executive function	0.34–0.79	0.64–0.72	0.54-0.75	0.52–0.88	0.24–0.51
Social cognition					
ToM	0.75–0.86				
Emotion recognition	0.35				
Emotion decision-making	Normal				

The range of values reflects heterogeneity in study samples but also heterogeneity because of the different neuropsychological tools used

It has been suggested that there is a deficit in working memory [45, 57, 72, 73, 77, 84–87, 126–129] and specifically in the visuospatial working memory [33, 130–134]. Psychotic patients might have worse performance [48, 135]. The impairment is probably present already since the early stages of the illness [136]. Some studies have shown that the impairment in working memory affects only patients with acute mania [53, 131, 132]. Comparing to patients with schizophrenia, the impairment in visuospatial working memory is less pronounced [115].

Additionally, there are studies indicating a deficit in declarative memory [137] and specifically in the semantic [138] and the episodic memory [48, 109, 139, 140], verbal learning [45, 47, 72, 86, 141–147] and this is true both for BD-I and BD-II [134, 148]. A deficit is also present concerning verbal memory [38, 41, 73, 75, 87, 102, 111, 143, 144, 147, 149–151], also during periods of euthymia [152]. One study, however, has shown that depressed BD patients showed greater impairments in verbal memory than the euthymic BD patients [90]. It has been suggested that the magnitude of the effect size of the verbal memory deficit is 0.7–0.9 [41, 139] and it is less pronounced in comparison to that seen in schizophrenia [153, 154] and cannot be explained by the attentional deficit [120]. Patients at early stages of BD have better performance in the total immediate free recall and in delayed free recall compared to patients at a later stage and to patients with schizophrenia. Additionally, concerning the ability to retain words learned, BD patients at a later stage and chronic patients with schizophrenia were more impaired than BD patients at early stage and patients with recent onset schizophrenia [155]. It has been also indicated that delayed free recall is worse in patients with BD compared to healthy controls [156].

There are impairments in total learning as well as short- and long-delay verbal recall, recognition, discriminability and learning slope [157], associative learning [158], implicit motor learning [159], immediate memory [134], delayed memory [34, 45, 72, 77, 84, 85, 94, 108, 160], non-verbal memory [161], visual memory [38, 43, 44, 74, 79, 102, 108, 111], autobiographical [162, 163] and prospective memory [164]. It has been shown that prospective memory deficits [165] and short-term non-affective memory [166] are also present in remitted BD patients suggesting that they constitute a trait deficit. Moreover, it has been shown that BD patients manifest a deficit in incidental contextual memory in the absence of a binding cue at encoding. There was no difference found between the groups for contextual memory even under incidental encoding with the binding cue. One study has indicated that the impairment in the contextual memory was reduced by providing cognitive support at encoding [167].

There are also negative studies concerning the presence of impairment in working memory [168–170], spatial working memory [171] and in verbal learning [138] and verbal [123, 172, 173] and visual memory [173]. One study has shown that the ability to learn is maintained both by BD patients and by patients with schizophrenia [155] and another study has reported that there were no differences between BD patients and healthy controls in terms of their slope of learning, retrieval index, retention percentage, semantic or serial clustering, errors, or level of retrieval [174]. It seems that most memory impairments are due to the presence of confounding variables except maybe for verbal recall [101]. The possibility that difficulties in the semantic clustering or other strategic processing deficits are the cause for the verbal memory impairment has both positive [139, 160] and negative data [137, 139, 161, 175].

In meta-analytic studies, when all phases of the illness are taken into consideration, the magnitude of the effect size is 0.60 for working memory, 0.43 for immediate verbal memory and 0.34 for delayed, 0.26 for immediate visual memory and 0.51 for delayed [65]. The meta-analysis of short-term memory studies revealed an effect size of 0.58 when span tasks were utilized, without any difference between auditory or visual tasks. When verbal learning tasks were used, the effect size was 0.91 [63].

During the acute manic/mixed state, the magnitude of the effect sizes was 1.43 for verbal learning and 1.05 for delayed free verbal recall. Additionally, concerning the acute depressed state, the effect size for verbal memory was 1.20, while during euthymia the domains impaired were working memory (0.65), verbal learning (0.81), long-delay verbal free recall (0.78), immediate non-verbal memory (0.73) and delayed non-verbal recall (0.80) [11].

The average effect size for episodic memory in euthymic BD patients is reported to be equal to 0.62 and for working memory equal to 0.60 [12]. Again in euthymic BD patients, a large effect size (0.90) is reported for verbal learning and working memory (0.98) and somewhat lower effect sizes for aspects of immediate (0.73) and delayed (0.71) verbal memory [13]. A third meta-analysis reported effect sizes of 0.81, 0.54, 0.74 and 0.72, respectively [67]. Another meta-analysis reported an effect size of 1.02 for working memory. 0.85 for delayed recall, 0.82 for immediate recall and 0.62 for visual memory [8]. Finally, a last meta-analysis gave an effect size of 0.85 for verbal learning, and 0.73 for verbal memory-early recall [9, 66]. It seems there is a publication bias especially concerning verbal learning and after correction for this, the effect size is attenuated (from 0.85 down to 0.66) [9].

Overall, the literature suggests that BD, irrespective of illness phase, is characterized by a severe deficit in the acquisition of new information, but not in the retention [41, 63, 161, 175, 176]. In spite of some opposing data, the most probable interpretation which derives from empirical studies is that the attention and concentration deficits impair the acquisition of information and learning, by disrupting the engagement of effortful processing which results in a shallow rather than deeper level of processing (e.g., acoustic rather than semantic) [137, 175, 177–186].

#### Verbal skills

The evaluation of verbal skills includes mostly the evaluation of verbal fluency. Although the literature has reported that verbal skills are impaired during all phases of BD [84, 87, 108, 129, 147, 149, 166, 169, 187–189], there are some studies reporting that this impairment is not present during euthymia [41, 52] or during the first mood episode [121]. One study has indicated that there are errors in speech during the acute manic state, and these errors are independent from the co-existence of an attentional deficit [106].

Concerning the magnitude of the effect size, it has been reported that it is small [169] and even smaller in comparison to the effect size that seen in schizophrenia [61], however, patients with psychotic BD have a higher effect size (0.68–1.73) [94, 117, 190]. Even smaller (below 0.50) effect sizes have been reported [63], while when all phases of the illness are taken into consideration, an effect size equal to 0.63 emerges [65]. Both letter fluency and semantic fluency are impaired during the acute manic/mixed state and the effect sizes are equal to 0.51 and 0.59, respectively. The phonemic fluency is impaired during the acute depressed state with an effect size equal to 0.93 while during euthymia impaired are both phonemic fluency (0.51), and semantic verbal fluency (0.75) [11]. In euthymic BD patients, the average effect size for verbal fluency ranges between 0.56 and 0.90 [12, 67] but it is smaller (0.34) for verbal fluency by letter [13].

#### Visuospatial skills

The evaluation of visuospatial skills is usually made with the use of the complex Rey figure or with the WAIS-R block design. Patients with BD and their unaffected relatives show impairment in the visuospatial/constructional abilities [45, 147, 191] and in visual learning and memory [191, 192]. It is interesting that euthymic BD patients and patients with schizophrenia have similar impairment when the results are controlled for possible confounding factors [193]. Also, one study has shown that BD patients were significantly impaired on all three object location memory processes (positional memory, object-location binding, and a combined process), with the largest effect found in exact positional memory (*d* = 1.18) [194]. Unaffected relatives demonstrated an intermediate level of performance in comparison to patients with BD and to normal controls [195]. Contrawise, one study suggested that the visual motion integration is intact in BD patients [196]. Some authors suggest that the impairment in visuospatial skills is restricted in the acute phase while these skills might not be affected during remission [41, 63]. One study has reported that the overall effect size is equal to 0.65 [63] and other studies have reported that in remitted patients the effect size is 0.22–0.57 [8, 12].

#### Executive function

The executive system is considered to be involved in the planning, the decision-making, the error correction and the troubleshooting, in situations where responses are not well rehearsed or contain novel sequences of actions, are dangerous or constitute technically difficult situations or situations requiring the overcoming of a strong habitual response or resisting temptation. In other words, ‘Controlling of mental and neurocognitive processes’ seems to be the key phrase describing the role of executive functions. In patients with BD, reasoning should be considered separately from the rest executive functions due to the fact that it seems to rely heavily on verbal and linguistic skills [63].

It has been suggested a severe impairment in executive functions except reasoning during all phases of BD [34, 36, 43–45, 48, 70, 72, 73, 75–77, 86, 87, 90, 94, 101–103, 108, 109, 111, 120–122, 129, 143, 146, 149, 190, 195, 197–209] and early during the course of BD [93], but the deficit is less pronounced in comparison to schizophrenia [38, 40, 61, 89, 116, 122, 210]. However, at least a subgroup of patients is as severely impaired as patients with schizophrenia [153, 211, 212]. One study indicates that the depressed BD patients showed greater impairments in executive functions comparing to the euthymic BD patients [90]. It is suggested that this impairment might be particularly severe concerning interference and inhibitory control [47, 80, 82, 108, 168, 190, 192, 213–215]. Additionally, patients with BD might have more risky [216] or erratic choices [217, 218] especially when a history of alcohol abuse exists [219].

However, not all data are straightforward. Normal overall executive function has been suggested by one study [144], while another reported only prolonged time to complete the test [207]. Also, a bimodal distribution of the Wisconsin Card Sorting Test (WCST) scores in patients with BD is reported, with some patients at near-control levels and others significantly impaired [41]. According to another study, the performance on the executive function measures is bimodal among euthymic BD patients (one subgroup with relatively normal and one subgroup with impaired executive functioning) [41]. Some authors argue that there is no difference between BD patients and controls in executive functions [78, 123, 170, 188, 220, 221], while others argue that the deficit is present only in the more severe and chronic cases [188]. It has been also indicated that BD patients show an improvement in the executive functions in the first year after resolution of their initial manic episode [91].

The deficit in executive functions might be overall independent from illness phase, however, there are data suggesting that some aspects of the deficit are related to affective lability [222], duration of illness, residual mood symptoms and current antipsychotic treatment [197] and history of psychosis [48]. One study found that the impairment in executive function was related to the severity of general psychopathology as it is measured by the PANSS [198] and another one correlated impaired insight with impaired executive functions [223].

A meta-analysis suggested that when taking all phases of the illness together, the effect size of this impairment is equal to 0.79 when reasoning (which was reported to be intact) is excluded [63]. Moreover, another meta-analysis reported an effect size of 0.34 for concept formation and of 0.55 for executive control, with no significant effect for current clinical condition [65]. During the acute manic/mixed state, the general executive function and the speeded set-shifting are impaired with the magnitude of effect sizes to be equal to 0.72 and to 0.64, respectively. During the acute depressed state, speeded set-shifting is impaired (0.64) while during euthymia problem-solving tasks (0.54), set-switching tasks (0.73) and verbal interference (0.75) are impaired [11]. Three recent meta-analytic studies in euthymic patients reported that the effect sizes were for executive functioning equal to 0.80 for the TMT-B, 0.56 for the WCST and 0.80 for the Stroop test [12] according to the first one, 0.78, 0.62 and 0.63, respectively, according to the second one [13] and 0.55, 0.69 and 0.71, respectively, according to a third meta-analysis [67]. Another meta-analysis reported an effect size of 0.99 for the TMT-B and 0.88 for the WCST perseverative errors and 0.52 for the WCST categories, 0.73 for the Stroop time and 0.65 for the stroop-correct [8]. Similarly, another study reported 0.70 for the WCST perseveration score and 0.8 for the TMT-B and the stroop test [9, 66].

#### Social cognition and theory of mind (ToM)

The term ‘social cognition’ constitutes a psychological domain with several dimensions. It refers not only to the ability of the person to assume that other people have minds similar to his/her own and to interpret, but also to understand and predict the emotions, desires, intentions, behaviors and speech of others (including non-verbal elements). Social cognition shapes communication and interaction with others and in this way enabling adaptive social adaptation. It involves a complex set of processes including the representation of internal somatic states, knowledge about the self, perception of others, and interpersonal motivations [224].

The broad theory of mind (ToM) includes three main processes (a narrow definition of ToM, emotion processing, and affective decision-making). The narrow definition of ToM (mentalizing or mindreading) refers to the ability to attribute mental states (e.g., beliefs, desires, and intents) to oneself and to others. Emotion processing is the ability to identify and discriminate basic emotions. Affective decision-making is crucial for an appropriate social behavior, and concerns weighing up choices in association with reward and punishment [225].

The tests which are used to evaluate these domains are both verbal (scenarios) and non-verbal (pictures). They demand the subject to identify and comprehend the situation, the roles and the interactions and to make appropriate planning. So far, empirical data have confirmed the universality of facial emotions. This means that the specific ability to process and identify facial emotions is a substantial feature of human communication and social interaction, which is independent of culture. It has been found, even in “ecological” tests that mimic real life scenarios that patients with BD showed less impairment on neurocognitive performance compared to patients with schizophrenia [154].

##### ToM

It has been shown that here is a deficit concerning ToM and social cognition during all phases of BD [162, 201, 216, 226–232], although there are also negative studies [233, 234]. It is possible that after controlling for medication and other confounding factors, the performance of patients is the same with the performance of controls [231]. There is also evidence that the impairment in ToM is restricted to the acute phases of the illness even when memory was controlled for [134] and thus there is no impairment during remission [235]. Moreover, patients with BD with psychotic features and patients with schizophrenia were found to be equally impaired in their scores for ToM stories. However, patients with schizophrenia manifested a worse performance [154, 236]. Another study reported no impairment in the accuracy of responses, but only the presence of a prolonged latency time for the response [237].

Generally, the literature supports the presence of a robust deficit in ToM in BD patients [78, 216, 232, 238–241]. This, in spite of one study which has shown that there are subtle deficits [229] and a second one which has reported no deficit at all [242]. Probably, the impairment in ToM in BD is associated with mood symptoms and it might reflect more fundamental underlying neurocognitive deficits rather than representing a specific trait marker of the disorder [134].

##### Emotion processing

There are inconclusive data concerning the recognition of emotions in BD patients. On one hand, there are studies indicating an impairment in emotion recognition, and in the identification and discrimination of emotions even during remission [193, 209, 229, 231, 240, 242–251]; on the other hand, however, there are studies reporting no impairment in these domains, especially after controlling for medication and other confounding variables [78, 231, 238, 239, 252–257].

Patients with BD I, seem to be more impaired in comparison to controls on face emotional recognition (FER) fear subtests, happiness, on the surprise test and on FER total scores [209]. It has been also reported that patients with BD do not have any impairment in face recognition in general, but the impairment exists specifically in the facial affect labeling, even during euthymia [246, 258]. Maybe specific phases affect specific emotions. For instance, in acute mania, the recognition of fear and disgust is reported to be impaired [247] while during euthymia patients recognize disgust better [259]. Reduced biases in the emotion recognition are related with acute bipolar depression while on the contrary increased biases in emotion recognition are related with acute mania [260]. This mood-congruent bias is state rather than trait and contributes as a core characteristic [245, 258, 261]. Younger BD participants performed worse than expected relative to healthy comparison participants of similar age. The deficits were found both concerning child and adult faces and were particularly strong for angry child faces, which were most often undertaken as sad. The results of this particular study were not influenced by medications, comorbidities/substance use, or mood state/global functioning [262].

##### Emotional decision-making

The literature reports that there is little or no difference between patients with BD and controls on the emotional decision-making component [207, 216, 218, 231].

##### Reviews and meta-analyses

One meta-analysis reported an effect size concerning ToM equal to 0.75–0.86 and concerning emotion processing equal to 0.35. The same analysis found that there was no difference between patients with BD and healthy controls concerning the emotional decision-making. That specific meta-analysis suggested that the performance in ToM and in emotion recognition was not associated with years of education, age, sex, duration of illness, and medication [225].

The significant heterogeneity in the results may be due to the differences among studies. These differences concern the neuropsychological tools used and the study samples. The specific tests might play a significant role since the performance of BD patients might be similar to controls in some aspects of emotion recognition but other aspects could be impaired or not, depending on the clinical state. For example, it has been found that stable BD patients might exhibit impaired facial emotion discrimination [193], while on the contrary the depressed BD patients’ performance could be similar to healthy controls in the perception of chimeric faces. In contrast, manic patients might perceive all chimeric faces as positive [263]. It is interesting that patients might outperform normal controls in specific domains (e.g., euthymic BD patients in the recognition of disgust) [259]. Depressed BD patients might show impairment only in the most difficult tasks [248]. It is important to be noted, however, that the effect of the clinical state is controversial since it is not consistent across studies [201, 216]. An additional problem is the fact that these are highly sophisticated neurocognitive functions and depend on the intact performance of lower ones; for example, the recognition of facial affect requires a compilation of attentional, executive and emotional abilities. Thus, it is difficult to be determined where exactly is the impairment located within these mechanisms [249].

In this frame, it is interesting that it has been found not only that the impairment in ToM is independent of other neurocognitive dysfunctions [201, 244] but also that this impairment is unrelated to a history of psychotic symptoms [227]. Of course, there are data supporting the opposite opinion as well, which seems more reasonable. Even in euthymic BD, executive dysfunction and some other neurocognitive impairments such as basic emotion recognition might be at least partially responsible for the impairment in ToM and social cognition tests [228].

A number of factor have been identified as contributing to of confounding the deficit. Low level of education and family history of BD might predict this impairment [216]. Moreover, the perception of emotion may be affected by the use of psychotropic medication and particularly by the use of benzodiazepines and both use and non-use of antidepressants. In healthy subjects, benzodiazepines impair the recognition of anger, citalopram and reboxetine reduce the perception of negative expressions and propranolol increases the reaction time to recognize sadness [216, 258].

Conclusively, although the available data suffer from significant methodological drawbacks, the literature suggests that there is an impairment in ToM in patients with BD [264]. Additionally, the theory that the deficit in emotional recognition occurs due to an impairment located in right hemisphere does not seem to be strong or sufficient [265] and any neurobiological dysfunction is likely to be state-dependent [266]

#### Clinical correlations

Neuropsychological dysfunction in BD may also be related to the clinical symptom pattern and severity, and it has been correlated with age, earlier age at onset, medication status, as well as with idiosyncratic factors affecting the long-term course.

##### The effect of medication

Medication constitutes an important confounding variable when comparing the different phases of BD. Some acutely ill patients might be medication-free during testing, however, this is not the case with patients in remission. As a result, medication status not only constitutes a confounding variable which is difficult to control for, but also might introduce a bias towards the detection of a deficit, especially in patients in remission. On the other hand, however, patients with severe mania or severe depression cannot be tested and are rarely off medication. Medication could be a possible reason why patients with BD have poor performance on certain neurocognitive tasks. This is in accord with the traditional concept that BD is considered to belong to the ‘functional psychoses’. According to this approach, the attentional impairment is considered to be the core neurocognitive deficit and the cause of all other deficits in neurocognition.

Overall, medication is considered to be an important factor, especially given also the possible neuroprotective or neurotoxic effect of several agents. For example, while most authors argue that lithium is neuroprotective, there is a possible neurotoxic effect in the long term, even at therapeutic levels, especially when it is prescribed in combination with antipsychotics [267].

It is of prime importance to differentiate the neurocognitive deficit caused by the illness itself and the deficit which could be medication induced. This differentiation determines not only the long-term therapeutical design but also the overall outcome. Moreover, such a differentiation requires a comprehensive assessment, on the basis of knowledge of those neurocognitive domains which are most affected by specific medication agents [268].

Patients under lithium often report that lithium inhibits their productivity and creativity [269]. There is no specific reason for this although it has been shown that lithium reduces unusual associations, and this could be the possible underlying mechanism [269]. On the other hand, however, it is not clear whether this constitutes a true deficit or it reflects a subjective feeling as a consequence of the transition from the manic/hypomanic to the euthymic state. It should be noted that this loss of creativity might be specifically related to lithium and not to divalproex [270].

Additionally, it seems that lithium has a negative impact on neurocognition especially on memory and psychomotor functioning [271–275] but fortunately the insult does not seem to be cumulative [276]. More specifically, it has been found that lithium impairs both mental and motor speed, short-term memory, and verbal or associative fluency, but the impairment is reversible when lithium is withdrawn and re-establishes when lithium is re-administered [272, 277, 278]. Lithium also causes a deficit in the long-term recall (retrieval) without having an effect on attention or on encoding [271, 278–281]. This impairment might especially concern verbal memory [94, 282]. Fortunately, it has been shown that cognitive complaints do not seem to be significant predictors of discontinuation of lithium treatment [283, 284].

There are limited data which disagree with the suggestion that lithium causes a significant neurocognitive impairment [285]. These data especially argue that lithium has no adverse effect on the reaction time [69] and executive functions [221]. Moreover, a longitudinal study found no evidence for a neurocognitive deterioration over a 6-year period in a sample of BD patients treated with lithium [276].

Overall, it seems that the effect size of the neurocognitive deficit related to lithium treatment is small and equal to 0.30 [8]. This is especially true concerning immediate verbal learning and memory (0.24), creativity (0.33) and psychomotor speed (0.62). Delayed verbal memory, visual memory, attention and executive function might not be affected at all [286].

The data on the possible deleterious effect of antipsychotics and antiepileptics on neurocognition are rare and conflicting [268, 287, 288]. Valproate and carbamazepine might cause an impairment in attention [289]. Topiramate, which is an agent which is not used in the treatment of BD per se, but is often administered in patients to treat a comorbid substance abuse disorder or to lose weight, impairs verbal memory, attention, causes psychomotor slowing, and impairs word finding even at very low dosages (25–50 mg/day). This deficit is reversible after discontinuation of the drug [277, 290].

In general, neuroleptics cause impairment in sustained attention and in visuomotor speed [291]. Moreover, even after controlling for clinical features, current antipsychotic treatment is related to worse performance across all executive function tests as well as in verbal learning and recognition memory and in semantic fluency in BD patients [41, 197, 292]. One study did not find any adverse effects concerning risperidone [293]. While another one suggested that years of exposure to antipsychotic medication were related to the impairment in executive functions [108]. It is unclear whether this deficit constitutes a true medication adverse effect or it is the consequence of the manifestation of psychotic symptoms, for the treatment of which, antipsychotics were prescribed.

Overall, it has been shown that medications have a limited adverse effect on neurocognitive function [294] if any at all [295–297]. On the contrary, there seems to be a close relationship between poor treatment adherence and neurocognitive impairment, but the causal inferences of these findings are uncertain. It is unclear whether it is the poor treatment adherence which leads to a worse neurocognitive performance through worsening of the overall course of BD, or, on the contrary, it is the neurocognitive impairment which causes poor treatment adherence and reflects a more severe form of the illness [298].

It is known that patients with BD are often treated with benzodiazepines which interfere with memory [299]. Also, some patients are treated with complex combinations of lithium, antipsychotics, antiepileptics, antidepressants, and benzodiazepines, and the combinatorial effects of these drugs on neurocognition are a matter of speculation rather than research.

Medication probably causes some degree of neurocognitive deficit particularly in sustained attention and in psychomotor speed [9]. It is difficult to differentiate this impairment from the impairment caused by the illness per se. It has been reported by studies comparing euthymic patients with or without medication that there are little effects of medication on neurocognitive test performance [104, 300]. It has also been mentioned that patients which were assessed during their first episode, thus before any exposure to medication drugs, also showed a neurocognitive impairment that was more or less similar, to the impairment observed in chronically medicated patients [301]. However, there is one study which reported that medicated BD-II patients performed worse in sustained attention than unmedicated BD-II patients [287].

In those individuals with full inter-episode remission doing well off medication, this adverse effect might be obvious, however it is also well known that staying off medication will adversely affect the overall course of the illness. It is unfortunate that no currently known pharmacotherapy improves neurocognition in BD substantially. Preliminary findings suggest some potential value for adjunctive stimulants such as modafinil and novel experimental agents [268].

##### The effect of psychotic symptoms

It has been shown that the presence of psychotic symptoms is strongly related to a worse overall neurocognitive performance [48, 57, 83, 84, 94, 100, 108, 129, 190, 227, 302–307] and in psychotic BD patients various aspects of the neurocognitive impairment are similar in magnitude to those observed in patients with schizophrenia [85, 133, 307]. Some negative studies exist as well [66, 77, 190, 227] and they suggest that the deficit in BD is overall less pronounced in comparison with schizophrenia. However, in several neurocognitive domains such as working memory and executive function, the deficit is similar to that seen in schizophrenia although as a general profile the neurocognitive deficit in psychotic BD patients is similar to that seen in unipolar psychotic depression [66].

A meta-analysis reported that patients with BD perform better than patients with schizophrenia, and the effect sizes of the difference varied between 0.26 and 0.63 for IQ, mental speed, verbal working memory, immediate visual memory, verbal fluency (with had the largest effect size equal to 0.63), executive control, and concept formation, but without any difference concerning the rest of domains [65]. It is important to note that there were only quantitative, and not qualitative, differences. Significant heterogeneity in effect sizes was present between studies, and this was partially due to the methodological issues and the size and clinical characteristics of the study samples [307].

Even in the absence of current psychotic symptoms, a history of psychotic features is also strongly related to a worse neurocognitive performance [129, 188], especially concerning measures of executive functioning, verbal and spatial working memory [48, 84]. However, it does not seem to exist a complete categorical distinction between psychotic and non-psychotic BD since this effect is modest [308]. The presence of family history correlates with a worse visuomotor attention in psychotic BD patients [309]; however, this load is less severe in comparison with schizophrenia since the offspring of mothers with BD has less neurocognitive impairment in comparison to offspring of mothers with schizophrenia [310]. Probably depending on the study sample (proportion of BD-I and BD-II patients) and the definition of psychosis, the results vary, and are suggestive of a nosological continuum between psychotic and non-psychotic bipolar cases. It is interesting that such a history of psychotic symptoms is inversely related to the neurocognitive function in the patients’ relatives [311].

Another meta-analysis reported that between patients with and without a history of psychotic symptoms, there were no differences concerning attention and visual memory. The observed differences in global IQ, mental speed, working memory, planning and reasoning and executive functions are small and only after excluding one outlier study [227], the effect size in the executive functions increases to 0.55 [308].

It is possible that the neurocognitive differences between psychotic and non-psychotic BD patients are in fact the result of an earlier onset of illness or medication use rather than a result of psychosis per se. In accord with this, one meta-analysis of cases suggested that psychotic BD patients in the above studies had a younger age of illness onset, more hospital admissions and a larger proportion of them were using antipsychotics. Also, they had lower education [308].

Schizoaffective patients perform poorer in a global way, maybe because current psychotic symptoms or history of psychosis is related to more severe neurocognitive impairment no matter the specific diagnosis [83, 129, 312]. Euthymic and stabilized schizoaffective patients are reported to perform worse than BD patients in attention, concentration, declarative memory, executive function, and perceptuomotor function [312, 313]

##### The effect of mood symptoms

Data are not available for very severely manic or depressed patients because most of these patients cannot be tested. Additionally, extrapolating conclusions on these patients from the study of less severe cases is problematic.

The overall severity of mood symptoms might not affect memory performance at least in those patients whose neurocognitive functions can be assessed [175]. There are studies showing no effect of either acute phase on the neurocognition of patients with BD [71, 142]. However, the bulk of the literature suggests that acute mania is associated to impulse control [314] and executive function impairments [315]. Acute bipolar depression is related with an attentional bias [287] with lowering of mental speed and impaired attention in general [100, 316]. There is also a verbal fluency [71], verbal recall and fine motor skills deficit [101]. It has been found impaired performance on theory of mind tests in both acutely depressed and manic patients, even when memory was controlled for [235]. The fluctuation in both manic and depressive symptoms have an appreciable impact on neurocognitive functioning [11]. While moderate changes in affective symptoms did not co-vary with neurocognitive ability [317] when changes are more severe or when rapid-cycling emerges, there is neurocognitive dysfunction [318].

It has been shown by a number of research studies and meta-analyses that in many domains there is a severe neurocognitive deficit even during remission [8, 9, 66, 307, 319], although there are also studies which keep some reservations [235, 320]. One meta-analysis showed that the illness phase had no effect on the short-term memory deficit [63] and some authors indicate that the consequence of antipsychotic therapy alone might be responsible for the impairment observed during the euthymic phase [292].

The data concerning the impairment in executive functions are inconclusive since there are studies suggesting that the deficit is independent from illness phase, however, there are reports indicating that some aspects of it are related to affective lability [222], duration of illness, residual mood symptoms, current antipsychotic treatment [197] and history of psychosis [48]. A relationship between the impairment in executive function with the severity of general psychopathology as it is measured by the PANSS [198] and with impaired insight have been reported [223]. Finally, differences in the neurocognitive performance could be the result of differences in somatic comorbidity (and co-medication) between BD patients and normal controls [321].

An important methodological problem is that the definition criteria for euthymia differ among studies and as a result, conclusions are suspect. Maybe even subsyndromal conditions might affect verbal memory [120] and also the presence of residual mood symptoms, regardless of polarity, might have a negative impact, on measures of attentional interference [197]. The impairment in ideation fluency is associated with residual mania, but not depression [285]. Our picture concerning the presence of the neurocognitive deficit during euthymia might change after controlling for residual symptoms in stabilized euthymic patients [19, 97, 192].

One study reported that apart from current symptomatology, the number of previous hospitalizations and family history of mood disorder are associated with the impairment in memory in patients with BD [137]. Particularly, the impairment in verbal memory might relate to the presence of subsyndromal mood symptoms, the duration of illness and the numbers of previous manic episodes, suicide attempts and hospitalizations [120, 144, 149].

The first follow-up study found that from patients without any neurocognitive impairment at first episode, one-third had significant deficits after 5–7 years [322]. The overall picture could be more complex since during the first episode of the illness, in non-psychotic patients, there might be no neurocognitive deficits at all, while, patients with psychotic features manifest a deficit comparable to that seen in patients with schizophrenia [302]. After the first episode, the time to recover was associated with executive function and possibly with verbal fluency [323].

A meta-analysis confirmed that neurocognitive impairment is present during all phases of BD. That meta-analysis calculated the respected effect sizes for specific neurocognitive domains separately for each phase. During the acute manic/mixed states, these effect sizes showed a clear impairment in attention (0.79–0.90), verbal learning (1.43) and delayed free verbal recall (1.05), letter fluency (0.51) and semantic fluency (0.59), general executive function (0.72) and speeded set-shifting (0.64). During acute bipolar depression, there were impairments in attention (0.80), verbal memory (1.20), phonemic fluency (0.93) and executive function in speeded set-shifting (0.64). During the euthymic phase, these effect sizes showed a clear impairment in auditory (0.41) and sustained visual vigilance (0.69) and speeded visual scanning (0.65), working memory (0.65), verbal learning (0.81) and long-delay verbal free recall (0.78), executive functions concerning problem-solving tasks (0.54), verbal interference (0.75) and set-switching tasks (0.73), immediate non-verbal memory (0.73), delayed non-verbal recall (0.80), visuospatial function (0.55), phonemic (0.51), and semantic (0.75) verbal fluency and finally in psychomotor speed (0.66) [11]. Overall, these results suggested that patients in a manic or depressed state had significantly greater effect size impairment in verbal learning than patients in an euthymic state.

The overall evidence suggests that the observed neurocognitive deficit in BD patients is not secondary and does not constitute a by-product of mood symptomatology or of exposure to medication. This is in spite of the observed strong relationship between mood symptoms and neurocognitive impairment. The most probable explanation is that neurocognitive impairment reflects a deeper neurobiological dysfunction which probably includes the presence of premorbid developmental abnormalities [324].

##### The effect of age and age at onset and personal psychiatric history

It has been well established that the overall progression of the illness causes and worsens the neurocognitive deterioration. The progression is a concept that cannot be easily defined and operationalized; however, there are a number of factors and indices which can be used to conceptualize it. These factors include the age at onset, the duration of illness and the number of previous episodes. A general belief is that the neurocognitive function is strongly associated with the severity of the disease [325].

*Age* The age of the study sample seems to play an important role, since young patients with BD have better performance compared to young unipolar patients, but the reverse is true in the elderly [326]. Overall, age seems to play a complex role. It has been suggested by one meta-analysis that there is significant impairment of neurocognitive function with advancing age [8], while a second one reported that the difference between patients with BD and healthy controls attenuates with age, and probably this happens because the neurocognitive performance of healthy people deteriorates with age, at a rate which seems to be faster in comparison to what is observed in patients with BD [12] thus leading to a floor effect. A similar phenomenon has been observed in schizophrenia [327–329]. Another meta-analysis reported no effect for age [66]

*Age at onset* The severity of the neurocognitive is correlated with age at onset [94, 330], especially in psychomotor speed and in verbal memory [9]. The early onset of illness and particularly the onset during childhood or adolescence is associated with more severe impairment [319]. In pediatric BD patients, the observed neurocognitive deficit is similar to the deficit seen in adult BD patients [331]. Especially, the impairment in attention is correlated with the age at onset [332].

*Personal anamnestic* During the course of the illness, the number of episodes [333], the number of prior hospitalizations [137, 188, 333, 334] and the longer duration [333] were associated with a worse neurocognitive function. Illness duration is related to the loss of inhibitory control [197] and to a general memory deficit [175] and verbal memory [149]. Also, worse neurocognitive function might be associated with the number of episodes but not with the duration of the illness [188, 275]. The impairment in attention is correlated with the age of first hospitalization and with the duration of the illness [332].

Some studies found that any history of mood disorder has an adverse effect on neurocognition and especially on memory [137, 175]. The number of past manic episodes, hospitalizations, and suicide attempts was correlated with more severe neurocognitive deficit [149]. It has been suggested that manic episodes were correlated with impairment in verbal learning and memory [144] and in attention and executive function [297], while the number of past depressive episodes was reported to have adverse effect on verbal memory [335] and reaction times [69].

The reverse explanation has also been suggested, with the neurocognitive deterioration being the cause rather than the effect of worse course and outcome [336]. The possibility that neurocognitive differences between psychotic and non-psychotic BD patients are in fact the result of an earlier onset of illness or current medication use rather than a result of psychosis per se cannot be excluded. In line with this, it has been reported that psychotic BD patients had more hospital admissions, a younger age at illness onset, and a larger proportion of them were using antipsychotics. They also had less years of education [308], which is something that should be taken into consideration since it has been suggested that education plays a significant moderatory role [11]. Finally, two meta-analyses suggested no significant effect for age at onset and duration or severity of illness, as defined by the number of episodes [9, 66].

##### The role of other clinical factors

There are many other clinical factors which could influence the neurocognitive performance of patients with BD. These include brain white matter lesions that are sometimes found in remitted BD patients and rarely in patients with schizophrenia but apparently do not underlie neurocognitive deficits per se [337]. Likewise, the lifetime comorbid alcohol use disorder, which also does not seem to correlate with the neurocognitive performance at least at earlier stages [316]. One study found that overweight and obese BD patients might have worse performance on verbal fluency [338].

Education could constitute an additional confounding variable, since patients with BD have lower educational level despite their IQ level which is comparable with that of controls. Thus, controlling for it might attenuate the magnitude of the observed neurocognitive impairment [339]. It has been suggested that the neurocognitive impairment effect sizes seem to decrease as a function of education [8, 12] and shorter duration of education is related to a more pronounced deficit in different domains such as letter fluency, WCST categories, and the stroop test [66]. The explanation might include two arms. The first concerns the possibility that education is a marker related to the onset and severity of illness, since early and severe illness interferes with educational attainment, and the second concerns the possibility that education is a protective factor per se.

#### Neurocognitive disorder in BD-II vs. BD-I

In spite of the research efforts during the last few decades, there has not been found a specific neurocognitive profile for the different bipolar subtypes [340]. This is probably because research on BD-II patients is rare, and there are large methodological differences between studies. As a result, inconclusive data are in place. Research on the other subtypes of the bipolar spectrum is essentially lacking. There is only one study reporting data on ‘bipolar spectrum’ patients. That paper suggested the presence of a broad neurocognitive impairment, particularly affecting verbal memory and the executive functions [341].

Some unsystematic reports in the literature suggest that BD-II patients have similar performance to controls [250, 342, 343], but others supported the notion that BD-II patients perform in-between healthy controls and BD-I patients [72, 73, 75, 79, 250, 344–346], but this maybe specific concerning verbal memory [149, 346] and executive functions [346]. On the contrary, there are data suggesting that BD-II patients perform similar to BD-I [72, 75, 138, 160], or even perform worse than BD-I patients, at least in some specific neurocognitive domains, including reaction time and inhibition [347, 348].

The literature cannot answer the question whether there is any qualitative difference between bipolar subtypes. Such a difference would suggest (although that would not be mandatory) that possibly there are different neurobiological mechanisms which underlie BD subtypes. However, both questions (concerning a quantitative and a qualitative difference) remain unanswered. For both there are data in favor and against.

A global neurocognitive impairment might be present in BD-II patients, with only phonemic verbal fluency being preserved and with moderate to strong effect sizes ranging between 0.62 and 1.34 [345]. For premorbid IQ, there is only one study which have not found any differences between BD-I and BD-II patients, since both groups had worse performance in comparison to the performance of controls [72]. It has been also shown that the intellectual decline was less pronounced in patients with BD-II in comparison to those with BD-I [347, 349]. There are several studies suggesting an impairment in psychomotor speed and in attention [72, 73, 287, 345, 346, 348]; however, other studies did not support this [342, 344]. One study suggested that reaction time was similar to that of controls [350]. Moreover, another study showed that attention was intact but psychomotor speed was impaired [79]. As far as memory, there seems to be a deficit in verbal memory and verbal learning [79, 149, 345–347] but some studies disagree [72, 73, 342, 344, 348]. Other authors found that in BD-II patients there is a presence of a less disorganized semantic system in comparison to patients with BD-I [138, 160]. Additionally, delayed memory is rather intact [72]. There are controversial data concerning the deficit in visual memory, since some authors reported an impairment [72, 345, 347] while others disagreed [73, 287, 342, 343, 348]. Similarly, some authors supported the presence of impairment in executive functions and in working memory [72, 79, 149, 344–348] while others did not [342, 343]. Additionally, there is one study which showed that there is a deficit only in working memory but not in executive functions [73]. It has been observed that all the studies using the stroop color–word test, which assesses interference, showed a deficit in inhibitory control in BD-II patients [344–347]. Also, there might be a deficit in the emotional processing domain [287, 347]; however, the emotion recognition seems to be intact [250]. It has been also found that unmedicated depressed BD-II patients had intact decision-making performance [343]. On the other hand, one study suggested that medicated BD-II patients had worse performance in comparison to unmedicated BD-II patients in sustained attention [287].

One meta-analyses suggested that there is no difference between BD-II and healthy controls neither in the estimated current intelligence quotient (IQ) nor the premorbid IQ [340, 351]. Another meta-analyses found that neurocognitive impairment in BD-II patients is as severe as in BD-I patients except for the domains of semantic fluency and memory [10]. There are contradictory data concerning psychomotor speed, verbal and visual memory and the impairment in these domains is probably small in magnitude. On the contrary, there are robust data concerning the presence of a working memory deficit and a decrease of cognitive flexibility and impaired inhibitory control in BD-II. Patients with BD-II manifest a deficit also in recognizing emotions [351]. In quantitative terms, BD-II does not differ much from BD-I [119] although it seems that the opinion which prevails is that BD-II patients manifest better performance in comparison to BD-I but worse than healthy controls and are positioned in between these two groups.

#### Long-term development of the neurocognitive deficit

##### Methodological issues

It is difficult to chart the long-term course of BD since there are no reliable indices to describe and chart the course in a global way. This happens because BD is a complex illness with different phases and clinical characters. Moreover, there is no clear direction of causality. One possibility is that the accumulation of mood episodes impacts negatively the neurocognitive function; however, the reverse is equally possible. A third possibility is that both the neurocognitive deficit and mood symptoms, independently from each other, reflect a specific pattern of clinical course and disease phenotype without any direct relationship to each other.

##### Premorbid period

Patients with BD have a relatively intact neurocognitive functioning throughout childhood and adolescence, and the neurocognitive impairment emerges only after the overt symptom onset, and this is in contrast to what is known concerning patients with schizophrenia [352]. In accord with this, it has been found that children who later develop BD exhibit good academic functioning prior to illness onset [32, 353–356].

However, some studies argue for the opposite. A prospective investigation of executive functioning in at-risk adolescents showed an impairment in executive function in those who later developed BD [357]. Also, an increased prevalence of abnormal developmental history has been shown with delayed language acquisition, and motor and social development in a group of adolescents with BD [358]. A large Finnish cohort study which evaluated verbal, arithmetic and visuospatial reasoning in healthy male conscripts (mean age 19.9 years) showed that the premorbid visuospatial impairment was associated with later development of both BD and schizophrenia [359]. Also, another prospective study from Sweden suggested that 7 % of 56 adolescents with developmental deficits at the age of 6 years, went on to develop BD, compared with none from the control group [360].

##### Early stages of BD

The neurocognitive maturation and the development of the child is probably adversely affected by the development of BD during childhood [361]. Generally, the overall neuropsychological deficit has been associated with earlier age at onset which, however, is unclear to which extend it represents simply a longer duration [330]. Immediately following illness onset, adolescents with BD exhibit poor performance in social and neurocognitive domains [353]. Similarly, in adults, the impairment is present already during the first mood episode [301]. At illness onset, there is a deficit in many domains such as sustained attention [97], spatial/non-verbal reasoning, learning and recall, and several aspects of executive function [362] as well as memory, verbal fluency and executive function [121].

##### Medium stages of BD

Patients with two or more illness episodes manifest poorer performance than patients with just one episode [275] and within 1–3 years, a significant further deterioration in the executive function can be observed [363], which along with processing speed are considered to be the main long-term neurocognitive deficits in BD [364]. One study found that the rest neurocognitive functions seem to be stable at 1–3-year follow-up [363, 365]. However, the complete picture of the results is controversial concerning the short-term deterioration [53, 147, 151, 153, 192, 334].

Latter in the course of the illness, repeated acute episodes negatively impact the neurocognitive functioning [352], which seems to correlate with both the number of affective episodes and the overall duration of illness [81, 97, 108, 144, 333, 366]. In turn, duration of illness and disease course are reported to correlate with verbal and visual memory and executive function [102, 108, 367]. The comparison of young, elderly, and chronic BD patients revealed that a greater number of chronic patients scored in the severely impaired range on a memory and executive battery than their counterparts with fewer past episodes [318]. Thus, it is possible that, instead of reflecting long-term damage to the brain because of repeated acute episodes, poor neurocognitive performance in multi-episode patients may be the result of the presence of chronic residual mood symptoms. It is known that BD patients experience mood liability [368, 369] and residual symptoms, usually depressive, during periods of euthymia in spite of the fact that they were rated as euthymic by clinicians [162].

##### Later stages of BD

Complex comorbidity is frequent in chronic patients and it is often accompanied with incomplete remission. Thus, the length of BD could act as a confounding factor in patients with comorbidity, e.g., with alcohol dependence in comparison to a non-alcoholic group [151]. A weak point in the literature is that although the acute effects of alcohol or drug intoxication have been controlled for in some studies [147, 176, 337], the effect of past exposures has not been taken into account.

Continuous medication treatment is also a confounding variable especially at later stages and when chronicity and complex comorbidity is in place. For example, although one study reported that there has not been observed further decline of neurocognitive function in patients under long-term lithium treatment [276], another study suggested that the executive function deficit was negatively correlated with years of exposure to antipsychotic drugs [108]. This latter finding could reflect either a toxic effect of long-term antipsychotic medication, the toxic effect of chronic psychosis or both.

In the long term, the neurocognitive deficit seems to be associated with the functional outcome. This relationship is particularly true for processing speed, attention, memory [370] as well as the visual/motor processing domain [371]. One study used cross-sectional data from a large case-register study of 14,000 people which were hospitalized with a mood disorder, 81,380 patients with osteoarthritis and 69,149 patients with diabetes. The results indicated that patients with BD have a 6 % increase in the risk of dementia with every episode leading to admission and overall this risk was higher in comparison to the two control groups [372, 373]. Although these data are in favor of a neurodegenerative process, it is also possible that the findings reflect different courses of the illness plus a different probability of long-term medication treatment and electroconvulsive therapy (ECT) with unknown long-term effects [123]. This effect has already been discussed since patients with more severe and frequent affective episodes perform more poorly on neurocognitive testing [333].

In accord with this, a course characterized by chronicity and residual symptoms with lack of remission between episodes is related to a progressive neurocognitive deficit and in this frame an important role is attributed to the specific clinical course of BD [318, 333]. As previously mentioned, there is a deleterious effect of psychotic symptoms that have deleterious effects [352] and as already previously mentioned antipsychotic medication is associated with poorer performance on IQ, memory and working memory assessments [46].

**Fig. 2:**
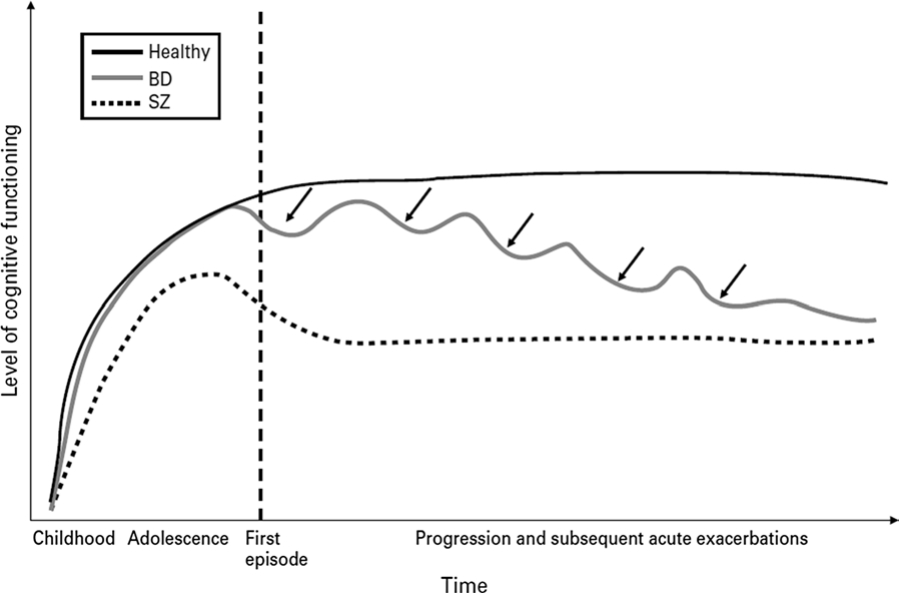
Long-term evolution of the neurocognitive deficit in BD patients, in comparison to patients with schizophrenia and normal subjects. Overall, in contrast to schizophrenia patients, BD patients exhibit a relatively intact neurocognitive functioning throughout childhood and adolescence, and the neurocognitive deterioration is observed only after the overt symptom onset. Reproduced after permission from Lewandowski et al. [352]

The overall longitudinal course suggests that neurodevelopmental factors play a minor role in the emergence of neuropsychological dysfunction in BD [324, 352]. Psychopathological factors during the course of the disorder itself are probably related to the neurocognitive impairment, but the nature of this association remains unknown. A graphical representation of the long-term development of the neurocognitive deficit in BD patients in comparison to schizophrenia and normal controls is shown in Fig. 2.

#### Awareness of the neurocognitive deficit

Although many patients with BD frequently complain about neurocognitive problems in attention, concentration and memory, there are limited data on the relationship between subjective cognitive complaints with objective neuropsychological deficits.

The patients’ subjective cognitive complaints do not seem to correlate or predict objective neuropsychological deficits [142, 164, 374]. One study has suggested that there might be a weak correlation between subjective complains and deficits in attention, memory and execute function [375]. Moreover, it has been shown that neither mood symptoms nor the severity of mania or depression correlate with patients’ self-report [142] One study reported that patients with BD might show more subjective complaints when there is a higher number of episodes, and especially a higher number of mixed episodes, a longer duration of the illness and when the onset of the illness occurred at an earlier age [375]. There seems to be some association between depressive symptoms and self-report neurocognitive complaints; however, the association between complaints and objective neurocognitive functioning is not moderated by mood symptoms [374].

#### Gender and the neurocognitive deficit

There is limited literature concerning the role that gender might play and no conclusions can be derived. Males with BD manifest a more severe deficit in immediate memory [376] and executive function [126] whilst one study suggested that gender does not play any role on emotion recognition [250]. A better memory performance might be related with female gender [175], while another study suggested that the verbal memory deficit might relate to female gender as an endophenotype [377]. Overall, the data are conflicting with one out of three recent meta-analysis being in favor of a gender effect [8], while the two others were against [12, 66].

#### The neurocognitive deficit as an endophenotype for BD

Endophenotype constitutes a stable pattern of behavioral symptoms with a clear genetic connection. It can be used to bridge the gap between high-level symptom presentation and low-level genetic variability. Ideally, an endophenotype might be useful in the differential diagnosis between disorders when they present with similar symptoms. Additionally, an endophenotype is associated with a specific disorder in the population, and should be heritable and state-independent. It is expected that endophenotype and the specific diagnosis co-segregate within families and the endophenotype is found in non-affected family members at a higher rate than in the general population.

The neurocognitive impairment in patients with BD is at least partially different in comparison to the deficit seen in patients with schizophrenia and it is also at least partially state-independent and definitely present during periods of complete recovery and euthymia. Although the literature so far does not support the use of global measures of neurocognition as endophenotypes [378–380]. On the other hand, however, specific neurocognitive deficits might be relevant, although further research is needed. It has been reported that inaffective relatives of patients occupy an intermediate position between patients and healthy controls concerning specific neurocognitive functions [195].

##### Twin studies

There is not much data concerning discordant monozygotic or dizygotic twins. Healthy co-twins performance in aspects of concentration, verbal and visual memory and verbal recognition and executive function is worse than controls [381], although a population-based study reported that the verbal memory deficit as an endophenotype might relate to female gender alone [377]. It has been also reported that neither BD patients nor their unaffected co-twins differed from control subjects in spatial working memory [171]. Another population-based study on healthy monozygotic and dizygotic twins showed that monozygotic high-risk twins manifested a significant deficit on selective and sustained attention, executive function, language processing and working and declarative memory, while the dizygotic high-risk twins manifested lower scores only on language processing and episodic memory [382]. No deficit was found on response inhibition in discordant twins of BD patients (event with a psychotic or familial form). Lower performance in twins during testing could be explained by current depressive symptoms [383]. It is interesting than in most of these studies, the unaffected co-twins suffered from minor or subthreshold mental disorders and dysthymia. Overall, twin studies provide some but not strong evidence for the presence of an endophenotype on the basis of neurocognitive impairment.

##### Studies on first-degree relatives

There are a limited number of studies in children whose parents suffer from BD. They suggest that high-risk children differ from control children in terms of reaction time; however, the quality data are rather low [384]. The same research group also reported that both high-risk and control children have similar performance in speech competence tests [385]. In two other studies, high-risk children were impaired in PIQ but not in VIQ; however, half of them were suffering from depression [28, 386]. Another study found that high-risk children and controls had similar IQ; however, controls had better performance in some reading and arithmetic cognitive tasks and there was an increased rate of VIQ-PIQ discrepancy [387]. A study on visual backward masking was also negative [69]. High-risk relatives had slower reaction time on a sustained auditory attention task [388]. Moreover, it has been shown by a recent study that specific executive functions which are supposedly located in the ventral frontal cortex might be impaired in high-risk children in comparison to controls [389]. Also, there might be a deficit in healthy high-risk children in spatial memory, in attention and in executive functions, even after correction for multiple confounding factors [390]. Overall, these studies provide evidence for the usefulness of executive function impairment as an endophenotype for BD.

##### Studies on mixed samples of relatives

There are more data concerning families of BD patients. These studies utilized mixed samples of first- and second-degree patients.

One study which utilized only female relatives of patients (most of them psychotic) reported that their IQ was superior to the IQ of controls. Also, it argued in favor of the usefulness of visual memory and executive function as endophenotypes [380]. Another study indicated that IQ was similar to controls [30] while on the contrary, another one reported the presence of a general and non-specific deficit in IQ, with some pronounced effect on verbal fluency [59]. More recent data suggest a higher discrepancy between VIQ and PIQ in families of patients with BD in comparison to healthy controls; however, these results were inconsistent with previous findings of higher VIQ in comparison to PIQ [356].

A deficit in psychomotor speed is also reported [52, 70, 143, 391], and while studies suggested an impairment in concentration [392], others have found that the impairment concerns shifting, but not sustaining attention (concentration) [98, 393, 394]. One study found a deficit in delayed recall in well-educated siblings with high IQ [395]. The literature also suggests the presence of an impairment in concentration, in the visuospatial declarative memory [392], in verbal working memory [396], and verbal [70, 143, 391, 397], visual [195] and auditory verbal learning [398] in working memory [399] and in executive function domain [52, 70, 143, 391, 396, 400].

There is one report on increased emotional interference with a bias towards mood-related information [401]. At-risk youths make more errors when identifying facial emotions, and the magnitude of the deficit was similar to that observed in patients with BD [243]; however, another study reported no deficit either in BD patients or in their first-degree relatives [257].

It is interesting that in spite of an overall normal neurocognitive functioning, there might be present some kind of subtle impairment in first-degree relatives of BD patients. A study which utilized l-tryptophan challenge showed that under challenge, a deficit emerges in relatives of BD patients in comparison to controls in memory, focused and divided attention, and psychomotor performance. Additionally, the relatives of patients with BD-I performed worse than the relatives of BD-II patients [402, 403].

##### Reviews and meta-analyses

Overall, the literature supports the idea that the deficit in executive function could constitute an endophenotype for BD, especially concerning psychotic cases. However, there are some data, which dispute the usefulness of specific executive functions, including cognitive control during episodic memory retrieval [397], response inhibition [383] and cognitive set-shifting [393]. Also, while a widespread deficit in attention and memory in families of patients with BD is reported, there are some negative data concerning concentration [98], psychomotor performance [396, 400], working memory [393], spatial working memory [171], verbal learning [391], verbal memory [396] and executive functions [400]. It is almost certain that IQ cannot serve as an endophenotype of the illness.

Reviews and meta-analyses suggest that the executive function and the verbal memory deficit could serve as core endophenotypes for BD [8, 367, 404], but conclusions are premature. The effect sizes reported are 0.49 with the stroop test and 0.37 with the TMT-B, while concerning immediate verbal memory the effect size is 0.42. Thus, in first-degree relatives, effect sizes were small (*d* < 0.5), but significantly different from healthy controls in particular concerning executive function and verbal memory [8].

In line with the above, another meta-analysis reported small effect sizes, that is, 0.20 for current IQ, 0.18–0.36 for attention and 0.17–0.22 for mental speed, 0.13–0.33 for various aspects of memory, 0.27 for verbal fluency and 0.24–0.51 for executive functions with the effect for the stroop test being the highest (0.51). This meta-analytic study demonstrated that impaired response inhibition might constitute the most prominent neurocognitive endophenotype of BD. Another executive measure, set-shifting (TMT-B, 0.38; WCST perseveration, 0.36) and two other neurocognitive domains, verbal memory (0.27–0.33) and sustained attention (0.36) also met the criteria as potential endophenotypes of BD. These results suggest that the response inhibition deficit, a potential marker of ventral prefrontal dysfunction, seems to be the most prominent endophenotype of BD [9].

In Table 2, the effect sizes of neurocognitive impairment in relatives of BD patients are shown. These effect sizes determine whether a specific impairment is of sufficient magnitude to be considered as an endophenotype.

## Discussion

Many areas of the neurocognitive function of BD still remain uncharted. It seems that a little less than half of BD patients suffer from a significant neurocognitive deficit and although the majority of BD patients might not differ from healthy controls, it is also true that some others do not differ from patients with schizophrenia. Both patient groups share a similar cognitive impairment profile with different degrees of deficits and thus the difference between them seems to be more quantitative rather than qualitative [405].

The complexity of the illness is evident also in the patients’ neurocognitive function and its assessment. The core deficit, which constitutes a direct consequence of the disease itself, seems to be independent not only from the other components of the disease but also from mood symptoms. This core deficit is either increased or on the contrary it is attenuated by many factors such as the disease phase, specific personal characteristics of the patients (age, gender, education, etc.), current symptomatology and its treatment and the long-term course and the long-term exposure to medication, psychiatric and somatic comorbidity and alcohol and/or substance abuse.

However, the origin and the etiopathogenesis of the core neurocognitive impairment remains elusive. Probably, there is a neurodegenerative component as a consequence of repeated mood episodes and psychotic features. Additionally, the effect of chronicity is quite strong while the neurodevelopmental component is either absent or very weak. This probably differentiates BD from schizophrenia, in which the neurodevelopmental component is strong. Such a neurodevelopmental effect is evident in some but not all patients with BP. A model suggesting that only BD patients who share common genetic risk factors with schizophrenia have premorbid neurodevelopmental neurocognitive deficits has been proposed [406]. On the other hand, there is no consistent evidence supporting that BD, as a group of disorders, have a progressively deteriorating course of neurocognitive functions [407].

Overall, the neurocognitive deficit concerns almost all domains with only a few exceptions and its magnitude is at the severe range during the acute episodes and at the medium range during euthymia. A summary of effect sizes by neurocognitive domain and illness phase is shown in Table 2.

There seems to be a small to moderate reduction in current IQ and general neurocognitive function during all phases of the illness; however, premorbid IQ and general neurocognitive function seems to be intact. Impairments in verbal memory, attention, and executive functions tend to be present during and after the first episode. Preliminary evidence suggests that these deficits in specific neurocognitive domains might precede the onset of illness [408]. The pattern and the severity of the neuropsychological impairment seems to be in place already during the first mood episode and these patients seem to lie intermediate between patients with a first episode of schizophrenia and healthy controls [409]

There is an impairment in psychomotor speed, attention, working memory and most of the verbal and some non-verbal components of memory as well as verbal fluency, executive functions and social cognition which is typically severe during the acute phases and moderate during euthymia. BD, irrespective of illness phase, is characterized by a deficit in the acquisition of new information, but not in retention, probably because of a disruption in the engagement of effortful processes. An important exception is the deficit in visuospatial skills which is present only during the acute phases and not during euthymia. An important methodological problem is that different studies utilize different definitions of euthymia and as a result, it is unclear whether subthreshold residual symptoms are present or not, what their polarity is and whether they are responsible for the deficit which is observed during remission.

The overall negative impact of medication is small and it is confounded by the specific clinical symptoms, for which medication is used for their treatment. This is especially true concerning antipsychotics and psychotic symptoms.

Although the prevailing opinion is that BD-II patients perform better than BD-I but worse than healthy controls, the literature has shown that in quantitative terms, BD-II does not differ much from BD-I.

The specific clinical course of the illness seems to play an important role. A course characterized by residual symptoms, chronicity and lack of remission between episodes is related to a progressive neurocognitive deficit. Of significant importance is the adverse effect specifically of psychotic symptoms, while manic episodes seem to affect neurocognitive function more than depressive episodes do. Age, age at onset, duration of the illness and number of episodes, not only reflect distinct but overlapping aspects of the overall disease burden but also are related to the neurocognitive impairment and its progression. However, the data are inconclusive concerning the magnitude and the true nature of this relationship.

The verbal memory and the executive function deficit could be the central endophenotypes of the illness. The most prominent endophenotype of BD seems to be the response inhibition deficit. However, in most family studies, unaffected at-risk family members who do not suffer from BD but they do manifest dysthymic or other, often subthreshold mood states, often in the form of temperament, and this might again act as a confounding variable.

## Conclusions

In conclusion, the literature suggests that the neurocognitive deficit in BD patients concerns almost all domains with only a few exceptions. Its magnitude is at the severe range during the acute episodes and at the medium range during euthymia, while the origin of the deficit remains unclear. In terms of neurocognitive function, BD patients do quantitatively better than patients with schizophrenia but the qualitative pattern of the deficit is similar in the two disorders. There are no clear differences between BD subtypes. The deficit is present early in the course of the disorder. At least in some patients, it might emerge before the onset of the first mood episode and in the majority of patients it progresses probably in relationship with the manifestation of psychotic symptoms. The verbal memory and the executive function deficit probably constitute endophenotypes, while the role of medication as a causative factor is limited.
